# High-Mobility Group Box 1 Induces Calcineurin-Mediated Cell Hypertrophy in Neonatal Rat Ventricular Myocytes

**DOI:** 10.1155/2012/805149

**Published:** 2012-06-20

**Authors:** Fei-fei Su, Miao-qian Shi, Wan-gang Guo, Xiong-tao Liu, Hong-tao Wang, Zi-fan Lu, Qiang-sun Zheng

**Affiliations:** ^1^Department of Cardiology, Tangdu Hospital, The Fourth Military Medical University, Xi'an 710032, China; ^2^Department of Biochemistry and Molecular Biology, The Fourth Military Medical University, Xi'an 710032, China

## Abstract

Cardiac hypertrophy is an independent predictor of cardiovascular morbidity and mortality. In recent years, evidences suggest that high-mobility group box 1 (HMGB1) protein, an inflammatory cytokine, participates in cardiac remodeling; however, the involvement of HMGB1 in the pathogenesis of cardiac hypertrophy remains unknown. The aim of this study was to investigate whether HMGB1 is sufficient to induce cardiomyocyte hypertrophy and to identify the possible mechanisms underlying the hypertrophic response. Cardiomyocytes isolated from 1-day-old Sprague-Dawley rats were treated with recombinant HMGB1, at concentrations ranging from 50 ng/mL to 200 ng/mL. After 24 hours, cardiomyocytes were processed for the evaluation of atrial natriuretic peptide (ANP) and calcineurin A expression. Western blot and real-time RT-PCR was used to detect protein and mRNA expression levels, respectively. The activity of calcineurin was also evaluated using a biochemical enzyme assay. HMGB1 induced cardiomyocyte hypertrophy, characterized by enhanced expression of ANP, and increased protein synthesis. Meanwhile, increased calcineurin activity and calcineurin A protein expression were observed in cardiomyocytes preconditioned with HMGB1. Furthermore, cyclosporin A pretreatment partially inhibited the HMGB1-induced cardiomyocyte hypertrophy. Our findings suggest that HMGB1 leads to cardiac hypertrophy, at least in part through activating calcineurin.

## 1. Introduction

Cardiovascular stress is often associated with the development of left ventricular hypertrophy (LVH), which is initially thought to be beneficial but can progress to heart failure if the stress persists [1]. LVH has been shown to be an independent predictor of mortality, and a major predisposing risk factor for the development of cardiovascular events in the native and transplanted heart; however, the underlying mechanisms are not fully understood [2–4]. Therefore, understanding the mechanisms involved in the pathogenesis of LVH is a critical step toward eliminating hypertrophy-related morbidity and mortality. 

High-mobility group box 1 (HMGB1) is an inflammatory cytokine, which is ubiquitously expressed in almost all types of cells; its proinflammatory properties mean that it has been linked to many inflammatory diseases [5–7]. HMGB1 can be actively secreted by activated immune cells or passively released by necrotic cells, including cardiomyocytes [8, 9]. It has been reported that the serum HMGB1 level in patients with endotoxemia, sepsis, hemorrhagic shock, rheumatoid arthritis, type 2 diabetes, hypertension, and ST-elevation myocardial infarction is significantly increased, and high serum HMGB1 level has been associated with pump failure, cardiac rupture, and inhospital cardiac death [10–15]. Furthermore, plasma levels of HMGB1 have been correlated with the severity of injury and tissue hypoperfusion [16]. It is important to note, that in the aftermath of the above-mentioned diseases, various conditions will trigger myocardial hypertrophy, and in turn induce deterioration of cardiac function [17]. Nevertheless, the direct effect of HMGB1 on cardiomyocytes, when serum levels are elevated, is not clearly understood. In this regard, recent studies suggested that Toll-like receptor-4 (TLR4) plays an important role in HMGB1 signaling, and that exogenous HMGB1 can cause myocyte contractile dysfunction via interaction with TLR4 [18–22]. Significantly, TLR4 signaling is reported to be responsible for the activation of calcineurin, leading to cardiomyocyte hypertrophy [23]. Given the incomplete understanding of HMGB1 function in LVH, in the present study we investigated whether HMGB1 contributes to cardiomyocyte hypertrophy, and whether HMGB1 induces hypertrophy via activation of calcineurin.

## 2. Methods

### 2.1. Cell Culture and Treatment

The experimental protocol was approved by the Institutional Care and Use Committee of the FMMU, which conforms to the EU Directive 2010/63/EU for animal experiments and the Guide for the Care and Use of Laboratory Animals of the US National Institutes of Health (NIH publication number 85-23, revised 1996). Monolayer cultures of neonatal rat ventricular myocytes (NRVMs) were prepared as described previously [24, 25]. Briefly, ventricular myocardium from neonatal Sprague-Dawley (SD) rats (aged 1d, The Fourth Military Medical University, FMMU) were homogenized and dissociated with collagenase II. Then the cell suspension was plated onto a 10 cm dish for 1 hour, enriched for cardiomyocytes by the technique of differential adhesion. The supernatant was then plated onto new dishes with Dulbecco's modified Eagle's medium (DMEM), containing 1% penicillin-streptomycin and 10% fetal bovine serum under 5% CO_2_ at 37°C. The remaining fibroblasts were minimized by the addition of 10 *μ*mol/L 5-bromodeoxyuridine for 24 hours. After another 24-hour starvation, NRVMs were then treated with recombinant HMGB1 (Sigma, St. Louis, MO, USA) for 24 hours. 

### 2.2. Measurement of NRVMs Protein Synthesis

The NRVMs were trypsinized and counted using a cell counting chamber (Beckman Coulter, Fullerton, CA, USA) and then lysed. The cell lysates were prepared to determine protein content by Bradford protein assay. Then the protein synthesis of cells was determined by dividing the total amount of protein by the number of cells, namely, protein per cell.

### 2.3. Quantitative Real-Time RT-PCR

Total RNA from NRVMs was extracted with TRIzol Reagent (Invitrogen, Karlsbad, CA, USA). RNA was quantified spectrophotometrically at 260 nm. A 20 *μ*L reaction mixture (Invitrogen) was used for reverse transcription. The reverse transcription products were then served as templates for PCR with gene-specific primers according to the previous report [26] (Table 1). Real-time PCR was performed using the 7500 Sequence Detector Real-time PCR system (Applied Biosystems, Foster City, CA, USA). The cycling conditions were as follows: 10 minutes at 95°C as an initial step, 15 seconds at 95°C, and 1 minute at 60°C for 40 cycles. Fluorescence signals of genes were recorded during every elongation phase of each PCR cycle. Melting curve analysis was used to confirm the amplification specificity. RNA abundance was expressed as ΔΔ*C*_T_. GAPDH expression served as the internal control. Each gene was quantified in duplicate. The data of real-time PCR were analyzed by the comparative *C*_T_ method as reported [27].

### 2.4. Western Blot Analysis

NRVMs were lysed in the lysis buffer containing 50 mM Tris, 150 mM NaCl, 1% Nonidet P-40, 0.25% superoxide dismutase, 1 mM EDTA, 1 mM NaF, 1 mM Na_3_VO_3_, and 1 mM phenylmethylsulphonyl fluoride. In addition, proteinase inhibitor cocktail (Roche, Basel, Switzerland) was added into the lysis buffer. The total protein samples were determined before being subjected to polyacrylamide gel electrophoresis and being transferred to nitrocellulose (NC) membrane. The NC membrane was immunoblotted with anti-ANP antibody (1 : 1000, Abcam), anticalcineurin A antibody (1 : 1000, Abcam), anti-ERK1/2 antibody (phosphorylated (p)- and total-ERK1/2, 1 : 1000; cell signalling, kindly provided by Dr. Yayun Nan), and anti-GAPDH antibody (Sigma; 1 : 10,000). GAPDH protein expression served as a loading control. Bands were evaluated by densitometry using the Odyssey infrared imaging system (LI-COR).

### 2.5. Calcineurin Activity Assay

The calcineurin phosphatase activity was measured by the dephosphorylation rate of a synthetic phosphopeptide substrate with a calcineurin assay kit (Enzo Life Sciences, Plymouth Meeting, Pa, USA) following the manufacturer's instructions. Then the released free phosphate was detected colorimetrically with the Green reagent on a plate reader at 620 nm.

### 2.6. Morphological Observation of Cell Size

NRVMs were fixed with neutral 10% formalin for 30 min and stained with 0.1% Crystal Violet (Sigma, St. Louis, MO, USA) for 10 min. Images were then obtained using inverted microscope.

### 2.7. Statistical Analysis

Data were obtained from three independent experiments and are presented as mean ± SEM. For the comparison between two groups, the Student *t*-test was employed. One-way ANOVA was used for multiple comparisons. A value of *P* < 0.05 was considered statistically significant.

## 3. Results

### 3.1. HMGB1 Induces NRVMs Hypertrophy

**Figure 1:**
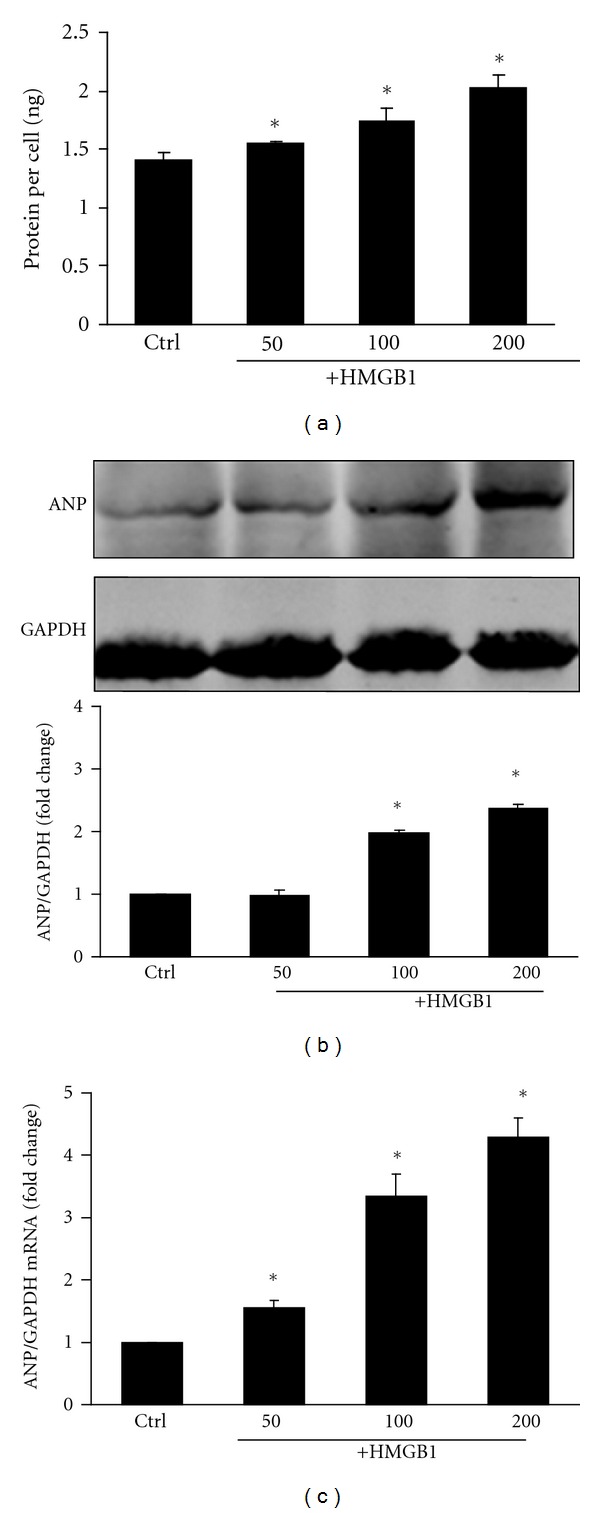
Effect of different concentrations of HMGB1 (50 to 200 ng/mL) on NRVM protein synthesis and ANP expression over 24 hours. (a) NRVM protein synthesis was determined by measuring protein content per cell. (b) Expression of ANP protein in NRVMs was determined by Western blot analysis, and the integrated optical density (IOD) of ANP expression bands was analyzed. (c) Expression of ANP mRNA in NRVMs was determined by real-time RT-PCR. The bar graph represents three independent experiments. *P < 0.05 versus control. Ctrl: control group.

Primary cardiomyocytes isolated from 1-day-old Sprague-Dawley rats were grown in culture and incubated with various concentrations of HMGB1 for 24 hours. At a concentration of 50 ng/mL, HMGB1 mildly increased cellular protein content by 9.3% compared with untreated control cells, while 100 and 200 ng/mL does of HMGB1 significantly increased the protein content per cell by 22.9% and 42.6%, respectively (Figure 1(a)). Given that pathological cardiac hypertrophy is characterized by the activation of fetal genes including atrial natriuretic peptide (ANP) [26], we analyzed ANP expression at the gene and protein level. HMGB1 treatment, particularly at the concentration of 200 ng/mL, significantly increased ANP protein and mRNA expression (Figures 1(b) and 1(c)). Taken together, these results suggest that HMGB1 treatment induced NRVM hypertrophy. 

### 3.2. HMGB1 Increases Calcineurin Activity in NRVMs

**Figure 2:**
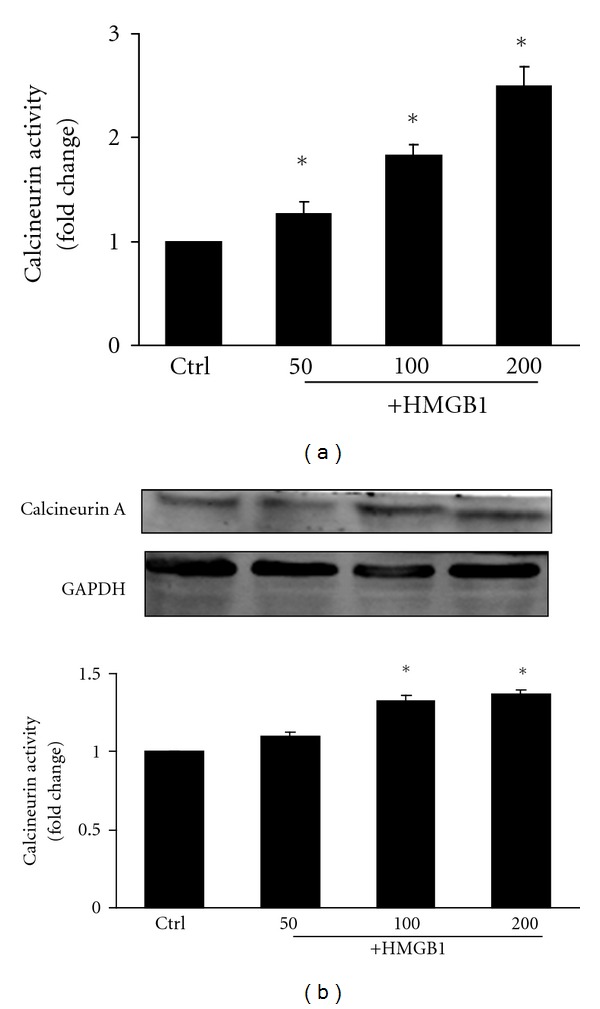
Effect of different concentrations of HMGB1 (50 to 200 ng/mL) on calcineurin activity and calcineurin A protein expression in NRVMs after 24 hours. (a) Calcineurin activity in NRVMs was determined by a calcineurin assay kit. (b) Expression of calcineurin A protein was determined by Western blot analysis, and the integrated optical density (IOD) of ANP expression bands was analyzed. The bar graph represents three independent experiments. *P < 0.05 versus control. Ctrl: control group.

Calcineurin activity was measured in HMGB1-treated NRVMs (Figure 2(a)). Incubation with HMGB1 was found to increase calcineurin activity, which was statistically significant at a concentration of 200 ng/mL. Calcineurin A is the catalytic subunit of calcineurin; expression of this protein was assessed by Western blot and found to be significantly increased with HMGB1 treatment at the concentration of 100 ng/mL and 200 ng/mL (Figure 2(b)). No significant difference in calcineurin A expression was observed between 100 ng/mL and 200 ng/mL HMGB1 treatment.

### 3.3. HMGB1-Induced NRVM Hypertrophy Was Inhibited by Preincubation with Cyclosporin A

**Figure 3:**
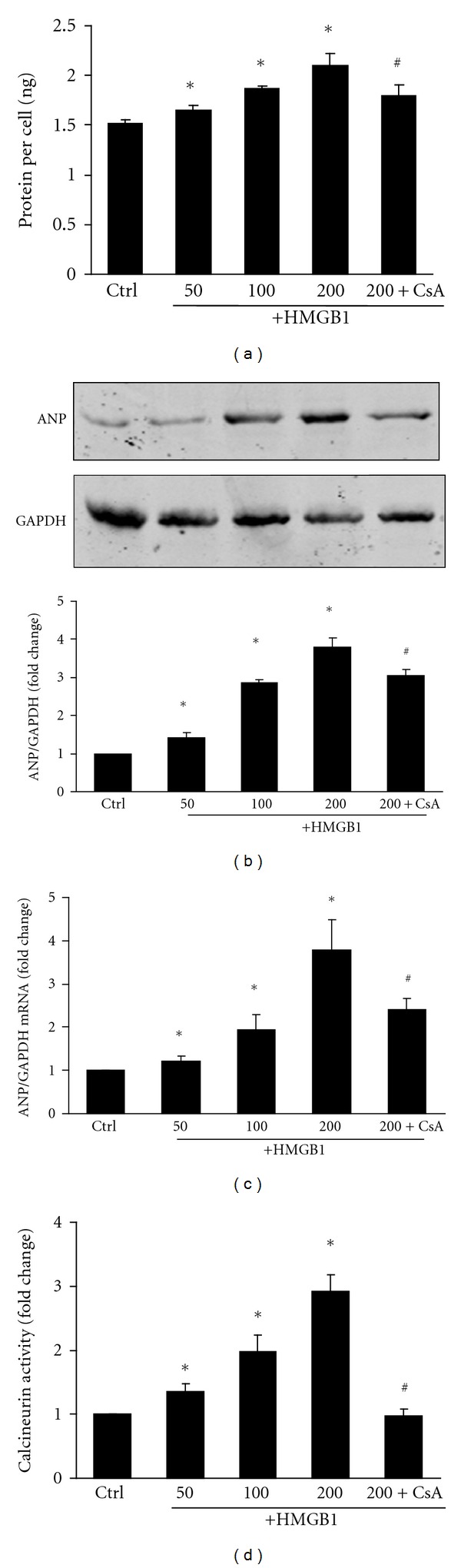
Effect of CsA on NRVMs pretreated with HMGB1. CsA (1 *μ*M) was applied for 1 h prior to a 200 ng/mL HMGB1 challenge in NRVMs. (a) NRVM protein synthesis was determined by protein content per cell. (b) Expression of ANP protein in NRVMs was determined by Western blot analysis; the integrated optical density (IOD) of ANP expression bands was analyzed. (c) Expression of ANP mRNA in NRVMs was determined by real-time RT-PCR. (d) Calcineurin activity was determined by a calcineurin assay kit. The bar graph represents three independent experiments. *P < 0.05 versus control. ^#^ *P* < 0.05 versus 200 ng/mL HMGB1 treated group. Ctrl: control group; CsA: cyclosporin A.

**Figure 4:**
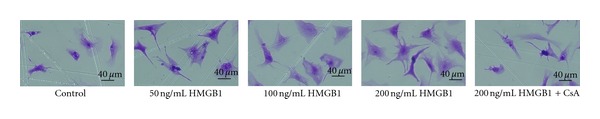
The cellular morphology change of NRVMs was observed using inverted microscopy.

Although our data indicated that HMGB1 significantly induced NRVM hypertrophy, in conjunction with an increase in calcineurin activity and calcineurin A protein expression, it was unclear if calcineurin directly mediated HMGB1-induced NRVM hypertrophy. To test this possibility, we applied 1 *μ*M of the calcineurin inhibitor, cyclosporine A (CsA), to our cell culture for 1 h prior to treatment with 200 ng/mL of HMGB1. Our results showed that CsA treatment partially inhibited the HMGB1-induced increase in cellular protein content (Figure 3(a)). Similarly, CsA pretreatment also attenuated HMGB1-induced expression of ANP (Figures 3(b) and 3(c)) as well as blocking the induction of calcineurin activity (Figure 3(d)). Analysis of NRVM cell morphology indicated an increase in cell surface area after treatment with HMGB1, which was inhibited by CsA (Figure 4). These findings suggest that HMGB1-induced cardiomyocyte hypertrophy was partially mediated by calcineurin.

### 3.4. ERK1/2 Expression in HMGB1-Induced NRVM Hypertrophy

**Figure 5:**
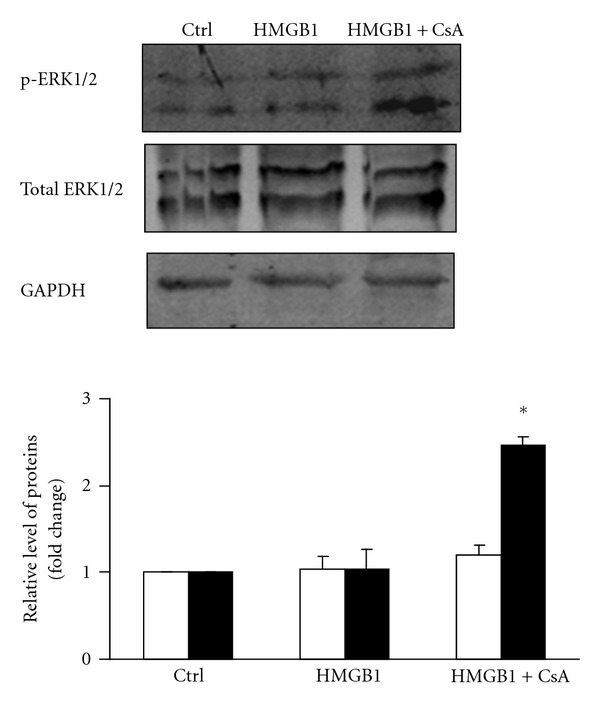
Effect of 200 ng/mL HMGB1 on ERK1/2 phosphorylation in NRVMs. NRVMs were pre-incubated with CsA for 1 h, and then incubated with 200 ng/mL HMGB1 for 2 h. ERK1/2 phosphorylation in NRVMs was determined by Western blot analysis. The graph shows fold changes in phosphorylated ERK1/2 (■) and total ERK1/2 (□). The bar graph represents three independent experiments. *P < 0.05 versus 200 ng/mL HMGB1 treated group. Ctrl: control group; CsA: cyclosporin A.

To test whether the hypertrophic actions of HMGB1 might occur through activation of ERK1/2, NRVMs were treated with HMGB1 and the phosphorylation of ERK1/2 was determined by Western blot. NRVMs were preincubated with CsA for 1 h, and then incubated with 200 ng/mL HMGB1 for 2 h. In the present study, HMGB1 alone did not stimulate the phosphorylation of ERK1/2; however, ERK1/2 appeared to be phosphorylated in the presence of both HMGB1 and CsA in NRVMs (Figure 5). 

## 4. Discussion

HMGB1 is an abundant protein present in cellular nuclei and cytoplasm; however, once released into the extracellular milieu, HMGB1 activates inflammatory responses, which can occur in inflammatory diseases such as sepsis and arthritis [12]. Numerous recent studies have also shown that HMGB1 acts as an inflammatory mediator in cardiovascular diseases, such as atherosclerosis [28–30], myocardial ischemia-reperfusion injury [9, 31, 32], and heart failure [33]. Most studies concentrated on the inflammatory effects of HMGB1, which works through the additional recruitment of inflammatory cells, endothelial cells, fibroblasts, cardiac c-kit^+^ stem cells, and extracellular matrix constituent degradation [34–36]. It is also suggested that HMGB1-induced inflammatory responses are mediated by infiltrated macrophages rather than the cardiomyocyte [9]. For example, HMGB1 stimulation of macrophages induced de novo synthesis of tumor necrosis factor-*α*, interleukin-1*α*, interleukin-1*β*, interleukin-6, interleukin-8, and macrophage inflammatory proteins -1*α* and -1*β*, all of which are important in the development of heart remodeling [37–39]. However, little is known about the direct effect of HMGB1 on cardiomyocytes without the help of other inflammatory factors. In the present study, we determined the effect of HMGB1 on hypertrophy status of NRVMs, and the possible mechanisms through which HMGB1 acts. We observed that HMGB1 increased the cellular protein content and ANP expression, two characteristics of cardiac hypertrophy. This finding is in agreement with works by Liu et al., who demonstrated that HMGB1 increases the capacitance of cardiomyocyte cultures [40]. 

**Figure 6:**
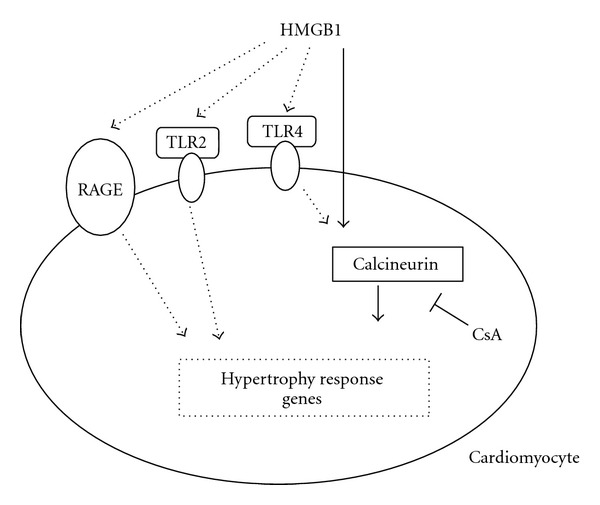
Proposed model for HMGB1 regulation of hypertrophy in NRVMs. HMGB1 induces hypertrophy associated with the activation of calcineurin, and this effect can be partially inhibited by CsA pretreatment. The solid arrows show the confirmative effect determined in this study, while the dashed arrows show the possible pathways to be determined. CsA: cyclosporin A; TLR2: Toll-like receptor-2; TLR4: Toll-like receptor-4; RAGE: receptor for advanced glycation end products.

Previous investigations have centered on identifying the molecular signaling pathways that regulate cardiac myocyte hypertrophy. One potential focal regulator of cardiomyocyte hypertrophy is calcineurin, which is sufficient to mediate cardiac hypertrophy and progressive heart failure [41]. HMGB1 has been demonstrated to transduce its signals by interacting with at least 3 receptors: receptor for advanced glycation end products (RAGE), TLR2, and TLR4 [9, 42]. Interestingly, lipopolysaccharide, a specific TLR4 agonist, leads to myocardial hypertrophy through the calcineurin signaling pathway [23]. Therefore, we determined whether cardiac hypertrophy induced by HMGB1 was mediated by the calcineurin-dependent pathway. In the present study, we observed that HMGB1 enhanced the activity of calcineurin. Furthermore, CsA pretreatment inhibited the HMGB1-induced increase in calcineurin and partially reversed hypertrophy. Therefore, our findings indicated that HMGB1 could precipitate the development of NRVM hypertrophy, in part through the activation of calcineurin (Figure 6).

The mechanism through which HMGB1 activates calcineurin is the focus of continuing experiments in our laboratory. It is known that calcineurin is a serine-/threonine-specific phosphatase that is uniquely activated by sustained elevation in [Ca^2+^] [43–45]. Interestingly, it has been demonstrated that HMGB1 impairs sarcomere shortening by decreasing calcium availability in feline cardiac myocytes, through modulating membrane calcium influx, acting as a novel myocardial depressant factor during cardiac injury [18]. These studies suggest that an HMGB1 induced decrease of calcium may contribute to the downregulated activation of calcineurin. However, given that intracellular calcium levels in cardiomyocytes change by 10 fold with every heartbeat and calcineurin activity might be chronically regulated, it is possible that calcineurin activity could be regulated through other mechanisms, such as increases in calcineurin protein levels, local changes in intracellular calcium pools, or through the actions of modulatory proteins [45]. In this regard, the present study also provided evidence that HMGB1 induces upregulation of calcineurin A protein expression, which may contribute to the observed increase in calcineurin activity. 

Recently, the mitogen-activated protein kinases (MAPKs) have been documented to regulate diverse biological functions including cell growth, differentiation, proliferation, and apoptosis. The MAPK signaling pathway is generally classified into three main branches, consisting of p38 kinases, c-Jun N-terminal kinases (JNKs), and extracellular signal-regulated kinases (ERKs) [25, 46]. The JNKs and p38 kinases generally serve as specialized transducers of stress or injury responses, hence their subclassification as stress-activated protein kinases (SAPKs), while the ERKs are somewhat more specialized for mitogenic and growth factor stimulation. The SAPKs function as mediators of dilated cardiomyopathy, while ERKs function as regulators of hypertrophy. It has also been demonstrated that SAPKs inhibit calcineurin signaling, whereas ERKs potentiate calcineurin activation [47]. Furthermore, ERK1/2 are thought to play important roles in the signaling hierarchy of cardiac myocytes [46, 48]. Similarly, transgenic mice overexpressing an activated MEK1 cDNA, which showed specific activation of only ERK1/2, were characterized by a prominent hypertrophic response [49]. It is known that SAPKs do not serve as forward regulators of the cardiac hypertrophic response, in contrast to the prohypertrophic regulatory role proposed for the ERK1/2 pathway [47]. In our study, there were no significant differences in the phosphorylation of ERK1/2 between control and HMGB1 treated NRVMs, though we interestingly observed that ERK1/2 phosphorylation was upregulated in HMGB1-treated NRVMs pretreated with CsA. Thus, the present study provides an intriguing observation that calcineurin activation, rather than ERK1/2, is a critical component of the prohypertrophic pathway through which HMGB1 works. Although CsA and HMGB1 cotreatment is likely to activate ERK1/2, which might potentiate calcineurin activation, CsA appears to contribute a major counteracting mechanism for HMGB1 induced hypertrophy via its direct inhibition of calcineurin. 

While our studies were performed in vitro, it is likely that hypertrophy in vivo is regulated by the balance of HMGB1 and multiple other inflammatory cytokines. Furthermore, HMGB1 is known to interact with various receptors, such as TLR2, TLR4, and RAGE, signaling through which can induce the activation of the nuclear factor-*κ*B [9, 50–53], HMGB1 has also been shown to potentiate TNF-*α*-induced JNK activation [54]. These signaling pathways all have the potential to contribute to cardiomyocyte hypertrophy in vivo and complicate the dissection of HMGB1's mechanism of action, necessitating continuing research in this area. 

In summary, our data demonstrated that HMGB1 induces cardiac hypertrophy, partially through activation of calcineurin. Thus, future research should establish HMGB1 as a promising target for treatment of cardiac hypertrophy in diseases such as endotoxemia, sepsis, hemorrhagic shock, rheumatoid arthritis, type 2 diabetes, hypertension, and ST-elevation myocardial infarction.

## Figures and Tables

**Table 1:** Primers for real-time RT-PCR.

Gene	Sense 5^′^-3^′^	Antisense 5^′^-3^′^
ANP	AGTGCGGTGTCCAACACAGAT	TTCTCCTCCAGGTGGTCTAGCA
GAPDH	TGCACCACCAACTGCTTAG	GATGCAGGGATGATGTTC
